# Interfacing Aptamer-Modified Nanopipettes with Neuronal Media and *Ex Vivo* Brain Tissue

**DOI:** 10.1021/acsmeasuresciau.3c00047

**Published:** 2023-11-22

**Authors:** Annina Stuber, Anna Cavaccini, Andreea Manole, Anna Burdina, Yassine Massoud, Tommaso Patriarchi, Theofanis Karayannis, Nako Nakatsuka

**Affiliations:** † Laboratory of Biosensors and Bioelectronics, Institute for Biomedical Engineering, ETH Zürich, Zurich CH-8092, Switzerland; ‡ Laboratory of Neural Circuit Assembly, Brain Research Institute, University of Zurich, Zurich CH-8057, Switzerland; ¶ Neuroscience Center Zurich, University and ETH Zurich, Zurich CH-8057, Switzerland; § iXCells Biotechnologies, Inc., San Diego, California 92131, United States; ∥ Institute of Pharmacology and Toxicology, University of Zurich, Zurich CH-8057, Switzerland

**Keywords:** biosensors, DNA, dopamine, fluidics, induced pluripotent stem cell-derived neurons, nanopore

## Abstract

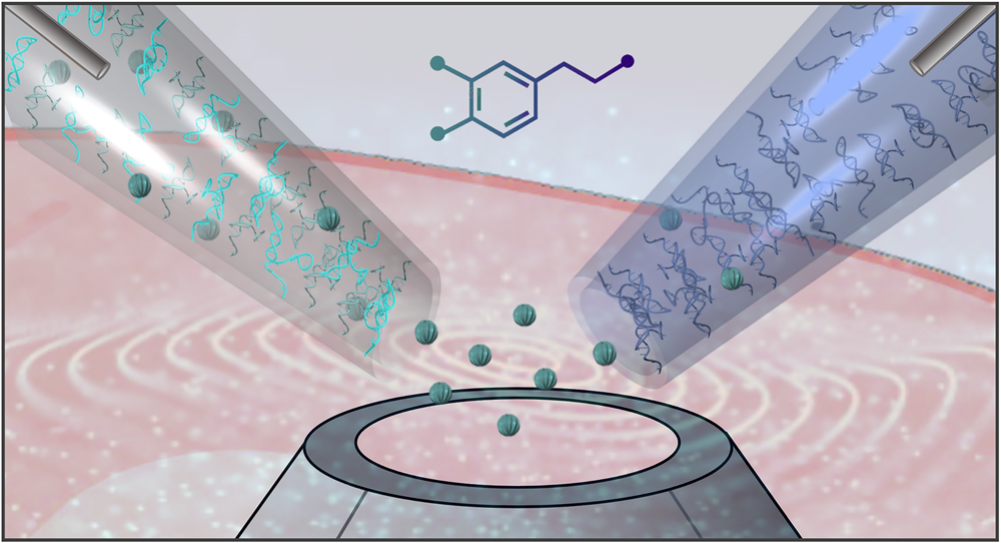

Aptamer-functionalized biosensors exhibit high selectivity for monitoring neurotransmitters in complex environments. We translated nanoscale aptamer-modified nanopipette sensors to detect endogenous dopamine release *in vitro* and *ex vivo*. These sensors employ quartz nanopipettes with nanoscale pores (ca. 10 nm diameter) that are functionalized with aptamers that enable the selective capture of dopamine through target-specific conformational changes. The dynamic behavior of aptamer structures upon dopamine binding leads to the rearrangement of surface charge within the nanopore, resulting in measurable changes in ionic current. To assess sensor performance in real time, we designed a fluidic platform to characterize the temporal dynamics of nanopipette sensors. We then conducted differential biosensing by deploying control sensors modified with nonspecific DNA alongside dopamine-specific sensors in biological milieu. Our results confirm the functionality of aptamer-modified nanopipettes for direct measurements in undiluted complex fluids, specifically in the culture media of human-induced pluripotent stem cell-derived dopaminergic neurons. Moreover, sensor implantation and repeated measurements in acute brain slices was possible, likely owing to the protected sensing area inside nanoscale DNA-filled orifices, minimizing exposure to nonspecific interferents and preventing clogging. Further, differential recordings of endogenous dopamine released through electrical stimulation in the dorsolateral striatum demonstrate the potential of aptamer-modified nanopipettes for *ex vivo* recordings with unprecedented spatial resolution and reduced tissue damage.

## Introduction

Almost five decades ago, a seminal paper by Ralph Adams demonstrated the importance of bridging the gap between analytical chemistry and neuroscience.^1^ Electroanalytical methods that could monitor neurotransmitters were applied to studying the complex neurobiology of the brain.^2^ In particular, electrochemical methodologies have focused on the detection of electrically oxidizable catecholamines such as dopamine, a neurotransmitter that plays a critical role in diverse clinical manifestations of mental dysfunction such as depression, schizophrenia, and Parkinson’s disease.^3^ While voltammetry was first implemented in the 1970s to track catecholamines,^4^ analyte selectivity was a critical concern due to overlapping redox potentials of similarly structured molecules such as dopamine and norepinephrine.^5,6^ Further, selectivity issues arose from interferents such as ascorbic acid that exist in significantly higher amounts in the brain vs catecholamines.^7^

Thus, until the late 1990s, microdialysis coupled to separation and detection methods remained the predominant method for measuring extracellular dopamine.^8^ Ongoing analytical advancements have expanded the capabilities of real-time dopamine measurements, specifically in the realm of fast-scan cyclic voltammetry (FSCV).^9^ Carbon-based electrodes with improved sensitivity, selectivity, and antifouling properties coupled to improved signal processing algorithms have been developed.^10^ However, the method still encounters challenges in selectivity with sensitivity limited to nanomolar concentrations.^10^ Further, FSCV electrodes suffer from signal degradation over time due to biofouling, where nonspecific molecules adsorb and encapsulate the sensing surface.^11,12^

We recently developed a novel electroanalytical methodology that tackles the remaining challenges such as selectivity, sensitivity, and biofouling encountered by conventional neurochemical detection methods. High selectivity is achieved through integration of artificial oligonucleotide receptors termed aptamers.^13−15^ These DNA-based recognition elements are traditionally isolated via advanced *in vitro* screening methods, enabling the detection of diverse small-molecule targets.^16−19^ Recently, neurochemical aptamers have been integrated into implantable transistor-based sensors.^20,21^ However, the dimensions of these microprobes are still on the order of hundreds of micrometers, sizes at which inflammatory responses and tissue damage may arise upon implantation.^22^ Further, for implantable sensors with surface-based detection strategies, direct exposure of the sensing area to biofluids leads to inevitable biofouling that inhibits long-term measurements.^20^

To address these challenges, aptamers that undergo conformational changes upon dopamine recognition^23,24^ were confined inside nanoscale pipettes (nanopipettes) with ca. 10 nm diameters. The rearrangement of the negatively charged dopamine aptamer backbone alters ionic flux through the nanopore, enabling target-specific signal transduction.^25^ The nanoscale opening not only improves the spatial resolution compared to existing microelectrodes or neuroprobes, but also limits exposure of the sensing surface to larger nonspecific proteins in complex milieu, reducing biofouling and increasing sensor stability.^26^ Confinement of sensing in a nanoscale volume theoretically renders our system sensitive to the presence of single to a few molecules.^27,28^ We demonstrate the applicability of this novel analytical nanotool for neuroscience by monitoring endogenous dopamine release from human stem cell-derived dopaminergic neurons as well as from the dorsolateral striatum of acute murine brain slices.

## Results and Discussion

### Sensing Dopamine Specifically in Artificial Cerebrospinal Fluid

**Figure 1:**
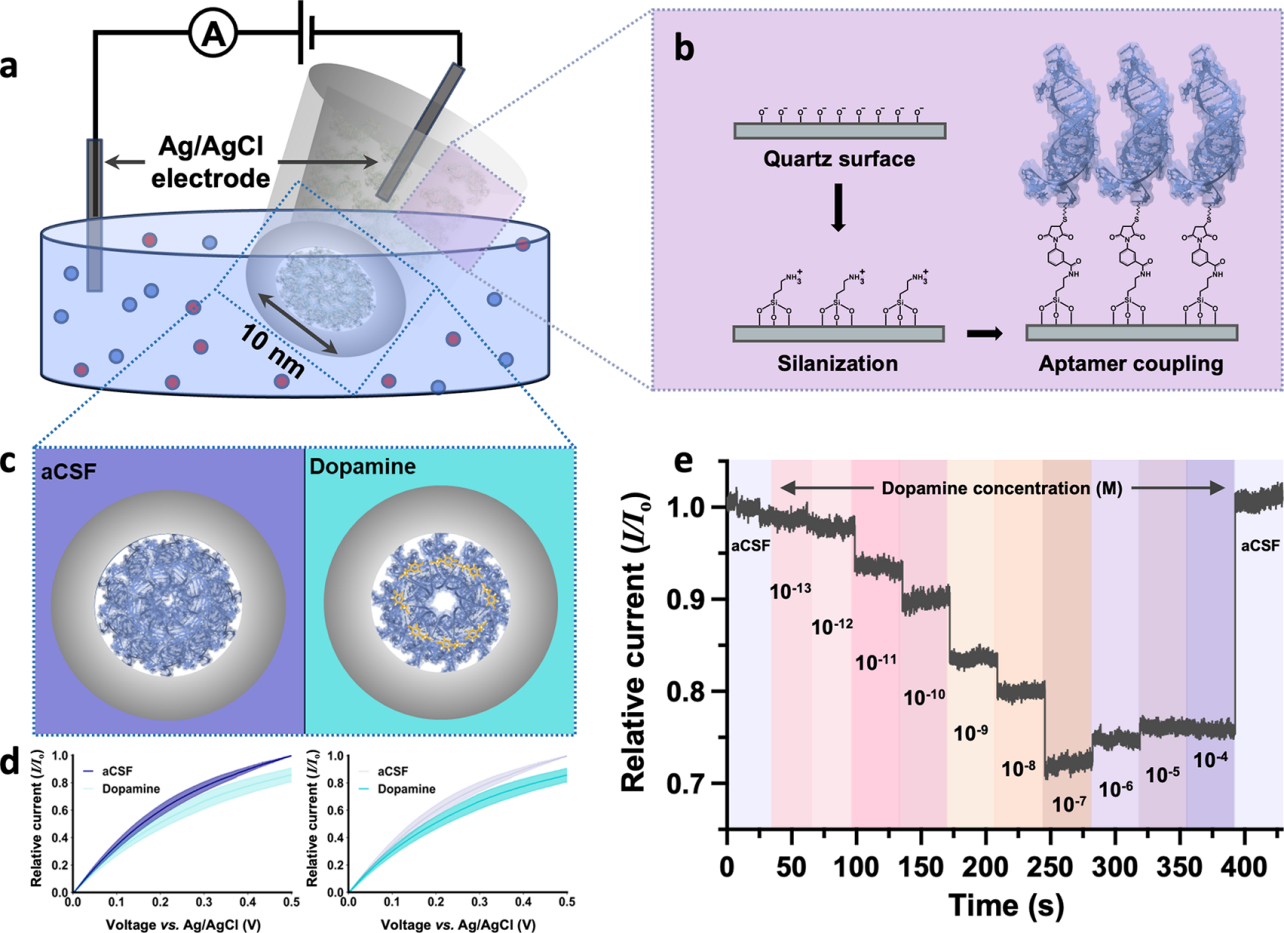
Dopamine aptamer-modified nanopipette mechanism and characterization in artificial cerebrospinal fluid (aCSF). (a) Schematic of dopamine-specific aptamers covalently modified on the inner surface of nanopipettes with ∼10 nm orifices. (b) Sequential surface chemistry to tether thiolated dopamine aptamers to the quartz surface. (c) Conformational changes of the dopamine aptamer upon target recognition alters the surface charge distribution within the nanoscale pore, altering the conductivity. (d) Current–voltage (*I*–*V*) sweeps in aCSF demonstrate a decrease in current response upon dopamine detection (100 μM, 14.2 ± 4.9%) relative to the respective baseline measurement (*I*_*o*_) in aCSF at 0.5 V. The average of *N* = 5 sensors is represented by the solid line with the standard error of the mean shown by the shading. (e) Concentration-dependent dopamine detection in aCSF, relative to the baseline obtained in aCSF. *I*–*V* sweeps are performed in each bath prior to the read-out, executed under a static potential. After conducting a sensor reset protocol using *I*–*V* cycles, the sensor returned to original baseline current values in aCSF.

Dopamine sensors were fabricated via covalent modification of dopamine-specific DNA aptamers inside the nanoscale orifice of nanopipettes with pore sizes of ∼10 nm (Figure 1a). The nanopipettes were filled with an ionic solution, and a Ag/AgCl electrode was placed inside, with a second electrode immersed in the measurement solution. Applying a voltage between these two electrodes induced ion migration through the nanopore, which was measured as an ionic current. Aptamer immobilization inside of the nanopore was achieved by first assembling monolayers of amine-terminated silanes on the quartz surface, which are subsequently coupled to thiolated aptamers. (Figure 1b).

The geometry of the nanopipette (conical shape and nanoscale pore) and the surface charges within the orifice resulted in a non-Ohmic behavior, leading to a nonlinear relationship between applied voltage and measured current, characterized as the ionic current rectification (ICR) effect.^25^ This nonlinearity allows the measurement of changes in ionic flux when the surface charge within the nanopore is modified, a mechanism exploited by structure-switching aptamers. In the presence of the target molecule dopamine, these aptamers reconfigure their negatively charged backbone within the nanopore, gating the ionic flux and altering the measured current response (Figure 1c).^29^ Prior to deployment in complex environments, dopamine sensors were characterized in artificial cerebrospinal fluid (aCSF) that mimics the ionic content of the brain milieu.

In aCSF, current voltage (*I*–*V*) cycles were conducted for the dopamine aptamer-modified nanopipettes. It is important to clarify that voltammetric sweeps are not conducted to detect dopamine via redox chemistry on the electrode surface as typically seen in FSCV. Instead, applied potentials serve to accumulate and concentrate dopamine inside the nanoscale pore for detection. This approach takes advantage of the single positive charge of dopamine under physiological conditions, enabling voltage cycles to facilitate the movement of the molecule toward or away from the nanopore, overcoming diffusion limitations. The sensing mechanism relies on dopamine aptamer-target interactions leading to changes in ionic conductivity at the nanoscale tip.^29^ An average decrease of 14.2 ± 4.9% was observed from the baseline current in aCSF upon addition of 100 μM dopamine (saturated concentration) when comparing the *I*–*V* curves at a voltage bias of 0.5 V (Figure 1d). The *I*–*V* curves decreased in a concentration-specific manner from baseline with increasing amounts of dopamine (Figure S1a), while showing minimal response to ascorbic acid (100 μM), which was added to dopamine solutions at 10 % weight per volume to hinder dopamine oxidation (Figure S1b).

The range in response magnitude is due to the inevitable sensor-to-sensor variability, which likely arises from slight geometrical variations in the laser-pulled nanopipettes, which influences the aptamer surface density within the sensitive region that spans ∼30 nm.^25^ To compensate for this variability, we characterize individual sensors prior to deployment as well as normalize the sensor to the current measured in the buffer void of any analyte. We have developed a protocol to facilitate sensor regeneration that is required for sensor reset, calibration, and subsequent reuse. Such a reset protocol is important for sensors that confine binding events inside nanoscale volumes. When target analytes encounter the mesh of aptamers fully occluding the nanopore, there is a higher probability of rebinding events to close proximity aptamers than target diffusion back to the bulk. Sensor regeneration requires repeated *I*–*V* sweeps in a dopamine-free environment to expel the trapped positively charged molecules from the aptamer-dense nanopore. Neglecting sensor resetting results in an altered receptor occupancy or aptamer availability for target recognition, which hinders reproducible repeated detection.

We demonstrate this reset protocol after sensors were used to detect dopamine under a constant applied bias of 0.5 V in aCSF. Starting in aCSF and subsequently varying dopamine concentrations from 10^–13^ to 10^–4^ M, a successive decrease in current response was observed until saturation was achieved (Figure 1e). Upon switching between different concentration baths, *I*–*V* sweeps were first conducted to accelerate signal stabilization, and then a static bias of 0.5 V was applied. Upon switching the buffer back to aCSF without dopamine present and conducting the resetting protocol, the original sensor baseline was achieved, demonstrating the resettability of the sensors after exposure to high dopamine concentrations. Real-time current measurements have been tested for multiple sensors (*N* = 3) over this wide range of concentrations to create calibration curves in aCSF (Figure S2). This reset protocol was further demonstrated for different sensors (*N* = 8, Figure S3a) and also multiple times by a single dopamine sensor (*N* = 3, Figure S3b).

### Determining Sensor Temporal Response in Flow System

**Figure 2:**
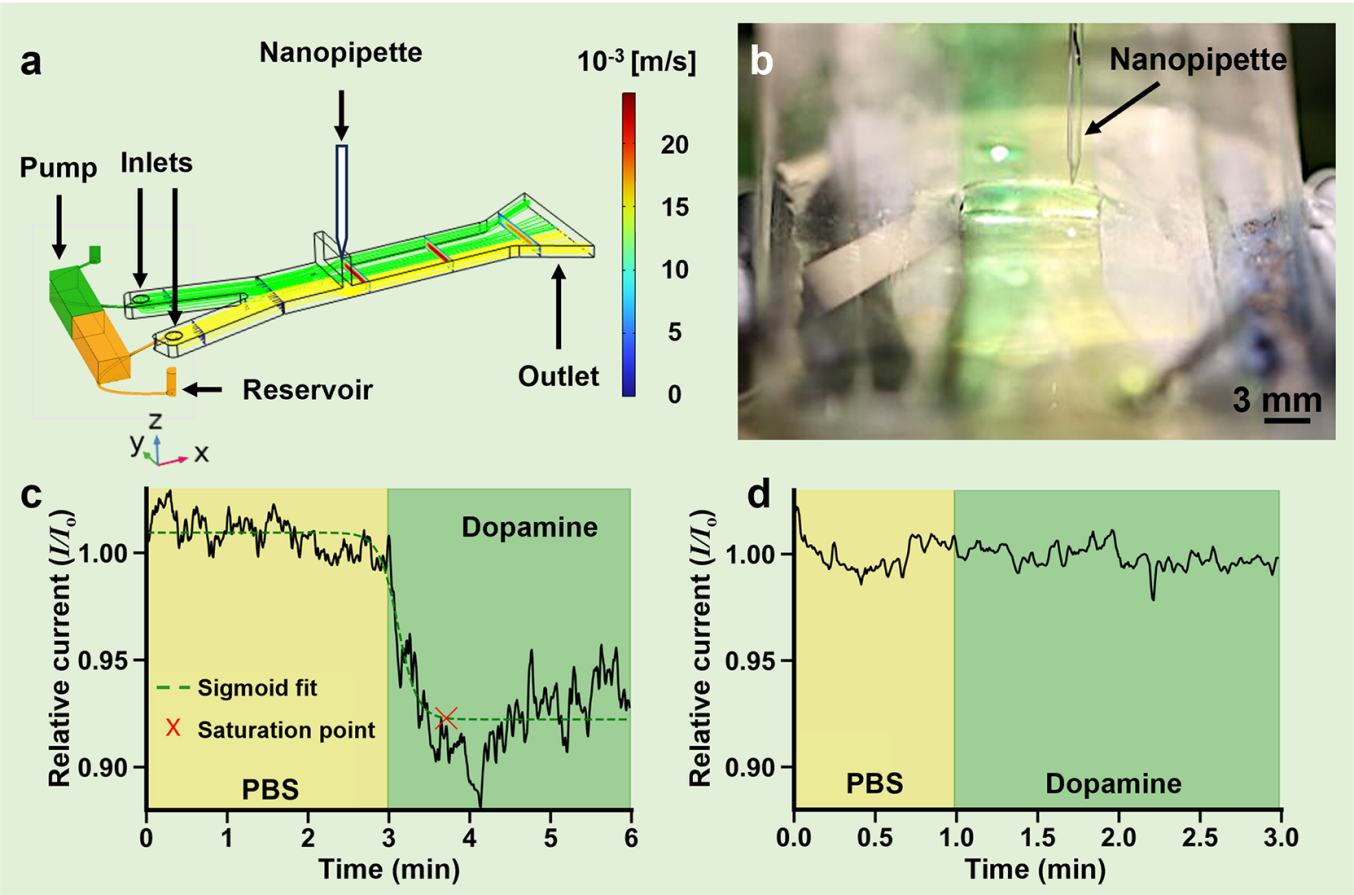
(a) COMSOL simulation of the pumped solutions in the macrofluidic channel with a slit in which the nanopipette is positioned. Phosphate-buffered saline (PBS, yellow) and dopamine (green) reservoirs are connected to a peristaltic pump, supplying both media into the fluidic channel. Streamlines of the two different liquids (laminar flow) are colored yellow and green. Fluidic velocity profiles are represented by the colored cross sections. (b) Photograph of a nanopipette sensor positioned in the PBS flow (yellow) through the measurement slit. (c) Real-time signal of a dopamine sensor measured in PBS flow (yellow) and the lateral transition to dopamine flow (green), represented relative to the baseline signal (*I*_*o*_) in PBS. A sigmoid was fit to the curve to calculate the point of signal saturation, used to determine the response time. (d) Normalized real-time signal of a control sensor transitioned from PBS (yellow) to dopamine flow (green) showing negligible changes in the baseline current, represented relative to the baseline signal in PBS.

Deployment of aptamer-modified nanopipette sensors in neuroscience applications demands real-time dopamine monitoring, and thus characterizing the temporal resolution is critical. A fluidic platform that facilitates rapid and clean switching of the solution to which the sensor is exposed was imperative to avoid temporal limitations imposed by the fluid switching process. To this point, we designed and developed a macrofluidic platform where two independent liquids are pumped in parallel with negligible mixing through a Y-channel made of polydimethylsiloxane (Figure 2a). A passive outlet allowed the liquid to spill into a collection container. We incorporated a slit into the channel to enable nanopipette sensor access to specific liquids. The flow profile and the macrofluidic design were optimized using COMSOL modeling to ensure liquids would not flow out of the measurement slit and remain laminar in flow.

The nanopipette sensors were lowered into PBS until visible contact with the liquid was observed to ensure optimal interaction with the laminar flow (Figure 2b). Food dye was used to visualize the positioning of the nanopipette in each flow (yellow for PBS and green for dopamine solution). Once a stable baseline was obtained in PBS, the micropositioner was used to transition the nanopipette laterally into the center of the neighboring dopamine flow. Upon interaction with dopamine (100 μM), the measured current of the sensor decreased instantly (Figure 2c). A sigmoid function was fit to the data to detect the point in time at which the signal saturated. While the sensor reacted immediately when entering the dopamine flow, the signal took between ∼2 min and ±45 s to reach 95% of the maximal signal change (*N* = 3).

The measurements were repeated with a control nanopipette to interrogate other nonspecific sources (*e.g.,* nanopipette movement, differences in ionic content between the two solutions) that may contribute to changes in the measured current. Control sensors were modified with scrambled DNA sequences that retain the same number and type of nucleotides as the specific aptamer but in an altered order, eliminating dopamine recognition. Scrambled sequences are optimal controls due to retention of sequence length and charge as the specific aptamer, which leads to comparable nonspecific interactions (*e.g*., electrostatic) that are inevitable in complex biological environments.^12^ The control sensor showed a negligible change when being moved from PBS to dopamine, confirming that the signal decrease observed for the dopamine aptamer-modified nanopipettes is specific to the presence of the analyte (Figure 2d). After characterizing dopamine aptamer-modified nanopipettes in buffer conditions, we transitioned to sensing in biological environments.

### Detecting Dopamine Release from Human Dopaminergic Neurons

**Figure 3:**
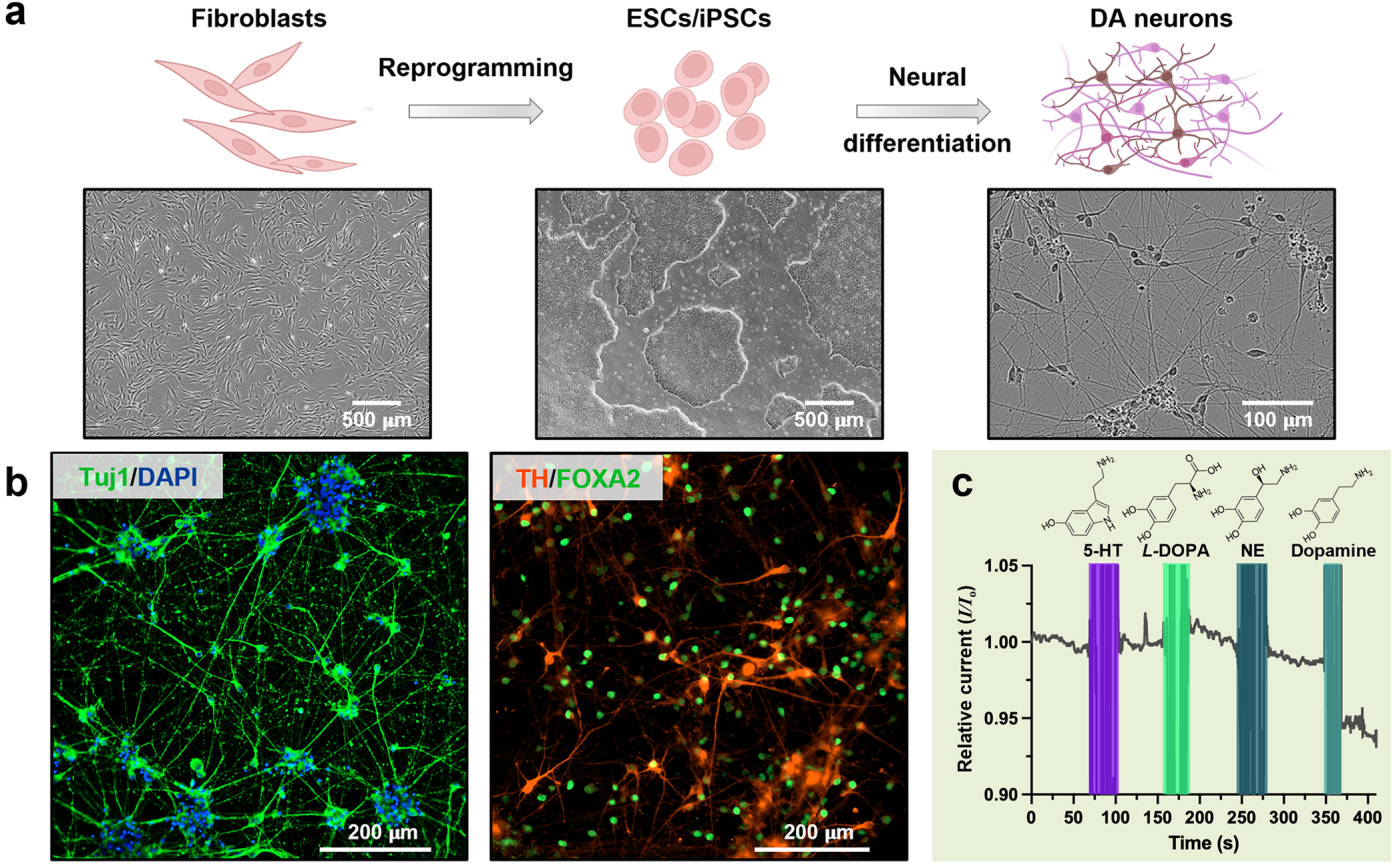
(a) Schematic representation of the process of obtaining human-induced pluripotent stem cell (iPSC)-derived dopaminergic neurons. Each step in the differentiation process was monitored using phase contrast imaging. (b) Human iPSC-derived dopaminergic neurons expressing characteristic biological markers. Immunostaining shows the expression of neuron marker neuron-specific class III beta-tubuli (Tuj1) and midbrain dopaminergic neuron markers forkhead transcription factor (FoxA2) and tyrosine hydroxylase (TH), 28 days post thawing. Nuclei were counterstained with 4′,6-diamidino-2-phenylindole (DAPI). (c) Dopamine sensors demonstrate selectivity in neurobasal medium with minimal sensor response upon injection of 100 μM analogously charged serotonin (5-HT), and structurally similar molecules l-3,4-dihydroxyphenylalanine (L-DOPA) and norepinephrine (NE) in real-time. A visible current decrease is observed only upon addition of 1 pM dopamine. The signal is represented relative to the baseline (*I*_*o*_) measured in neurobasal media.

Dopaminergic neurons have been generated from human-induced pluripotent stem cells (iPSCs) through various protocols.^30−32^ However, technical improvements are necessary to increase the cell purity for homogeneous cultures, improve reproducibility, and supply a sufficient quantity of dopamine-specific neurons. To tackle this challenge, iXCells Biotechnologies, Inc. have developed proprietary methods to reprogram fibroblasts obtained from healthy human subjects to embryonic stem cells and iPSCs. The fibroblasts were differentiated into dopaminergic neurons and each step of the process was tracked via phase contrast imaging (Figure 3a). This protocol gave rise to fully differentiated and functional human iPSC-derived dopaminergic neurons that display typical neuronal morphology and have high viability for over 60 days, despite freeze–thaw cycles.

Further, the iPSC-derived dopaminergic neurons expressed neuronal markers such as neuron-specific class III beta-tubulin (Tuj1) and key markers indicative of mature dopaminergic neurons, including tyrosine hydroxylase (TH) and the forkhead transcription factor (FoxA2) when cultured in Human Dopaminergic Neuron Maturation Medium (Figure 3b). To quantify the specific populations of dopaminergic neurons, TH-containing cells were counted using flow cytometry analysis after being labeled with antibodies (Figure S4). The dopaminergic neurons exhibited a high population (95.85%) of TH-positive cells 28 days post thawing. An important confirmation of functional and mature neurons beyond quantification of cells expressing specific markers, is to validate dopamine release. Such measurements are often nontrivial due to high ionic milieu containing nonspecific interferents. Selectivity tests vs structurally similar (l-3,4-dihydroxyphenylalanine, norepinephrine) and analogously charged (serotonin) interferents were performed in neurobasal medium that contains nonspecific proteins and amino acids for *in vitro* neuronal growth (Figure 3c).

**Figure 4:**
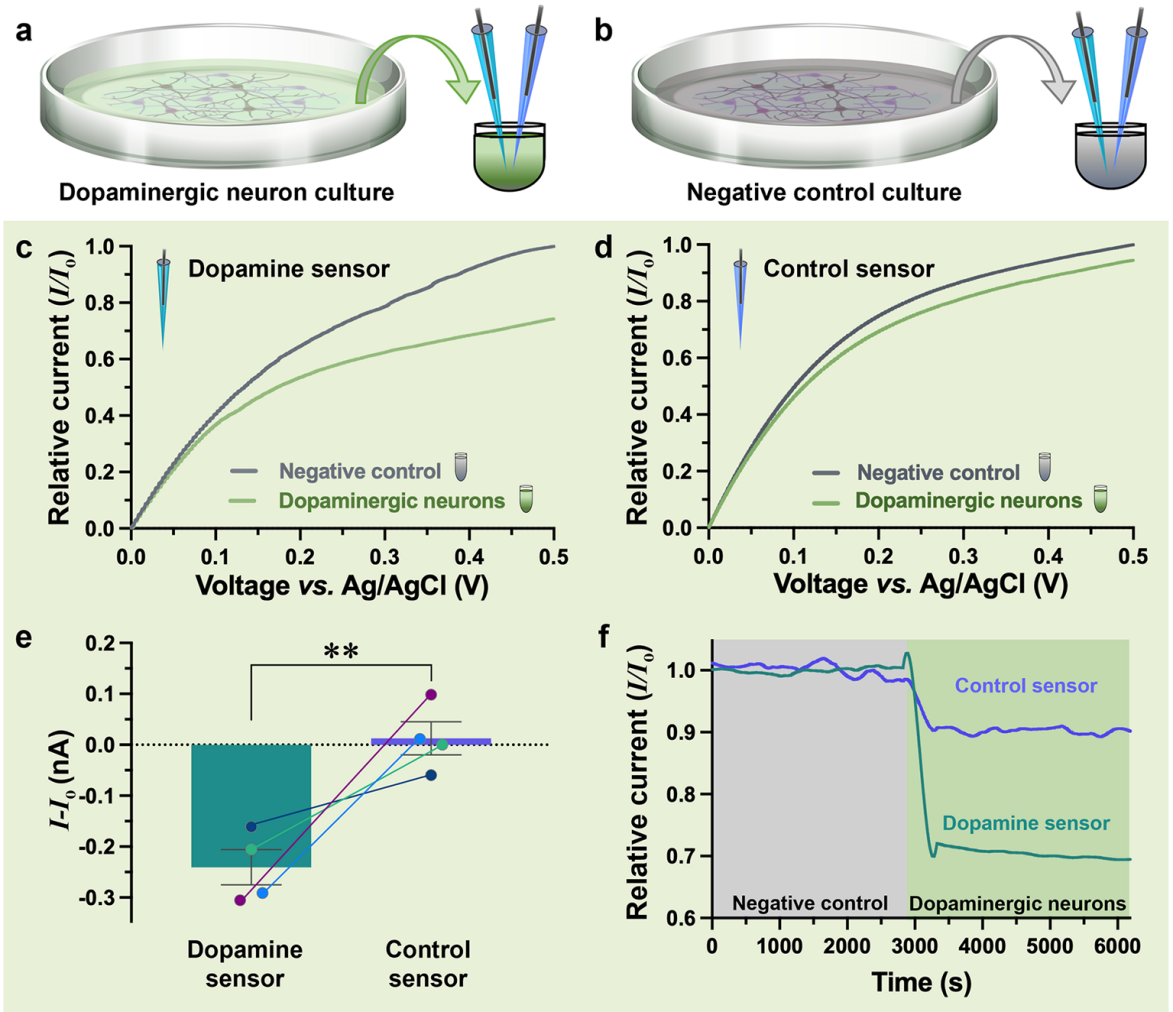
(a) Schematic representation of the cell medium harvested from dopaminergic neuron cultures and (b) negative control cultures with motor neurons that do not release dopamine. Both the dopaminergic (green) and control (gray) media are measured with a dopamine sensor (turquoise) and control sensor (purple). (c) Representative Current–voltage (*I*–*V*) curves of the dopamine sensor tested in different media, represented relative to the negative control. A clear decrease is observed in dopaminergic neuron media in comparison to the negative control media. (d) Representative *I*–*V* curves of the control sensor modified with a scrambled sequence showed smaller changes in current response between the dopaminergic neuron media relative to negative control vs the dopamine sensor. The difference in current values is likely due to differences in ionic content of the media. (e) Dopamine sensor (−0.24 ± 0.034 nA) detected statistically higher changes in current vs the control sensor (0.013 ± 0.033 nA) when comparing *I*–*V* readouts at 0.5 V in the dopaminergic neuron media vs negative control media [unpaired *t* test: t(6) = 5.341, *P* = 0.0018]. Lines connecting dopamine and control sensors represent recordings in the same sample. (f) Real-time responses of the dopamine-specific and control sensors from the negative control to dopaminergic neuron media. The difference in response between the two species indicates the specific dopamine response. The pair of sensors for which the control sensor had the highest response in the dopaminergic neuron media is shown to demonstrate the importance of conducting differential measurements. Signals are reported relative to their baseline measurements (*I*_*o*_).

From iXCell Biotechnologies, Inc., we received three samples: neuronal media in which dopaminergic neurons were grown for 30 days (Figure 4a), media collected from 7-day cultures of motor neurons (negative control neurons that do not release dopamine, Figure 4b), and pure neural differentiation media devoid of any cell contact. The three samples had different colors, indicative of differences in pH that were measured as 7.42, 7.84, and 7.75 for the dopaminergic neuron media, negative control media, and pure media, respectively (Figure S5). During differentiation and growth of dopaminergic neurons, pH changes occur due to metabolites released by the cells.^33^ As a different pH likely yields different ionic contents, which influences our measurements, we were unable to use the pure neurobasal medium as the comparative point to validate the presence of dopamine in the samples in which human dopaminergic neurons were cultured.

Differential measurements were conducted where specific dopamine sensors were tested in parallel with control sensors. This approach of differential sensing differentiates specific vs nonspecific influences, to changes observed in the baseline current. In situations where the physical environment changes (*e.g.,* pH), both the sensor and the control nanopipettes would exhibit a comparable baseline shift. Alternatively, when the change in signal results from specific molecular interactions, the sensor would register a signal while the control sensor would maintain a stable baseline. When testing the dopamine-specific sensor, a decrease of 24.3 ± 3.6% was observed in the *I*–*V* measurements conducted in the dopaminergic neuron medium normalized to the negative control medium (Figure 4c). On the contrary, when testing the control sensor, significantly smaller changes were observed (4.2 ± 2.3%) between the two samples (Figure 4d). Theoretically, the two media should show negligible differences when testing with the control sensor; the lower control sensor signal in the dopaminergic neuron media vs in the negative control is likely due to differences in ionic content, demonstrating the importance of having a reference sensor for comparative measurements.

The change in current response between *N* = 4 different dopamine and control sensors showed statistical significance (Figure 4e). Real-time measurements moving from the negative control medium into the dopaminergic neuron medium further enable visualization of the difference in response between the specific vs control sensor (Figure 4f). Thus, we demonstrated that the human dopaminergic neurons developed by iXCell Biotechnologies, Inc. release dopamine, retaining chemical functionality despite freeze–thaw cycles. To quantify the precise amount of dopamine in the human dopaminergic neuron samples, additional standard addition experiments were needed but unfeasible due to the limited sample quantity received for this study. Nevertheless, we demonstrated the ease of measurement of dopamine directly in neuronal media without experiencing clogging issues over hours of testing. In contrast, bare, unmodified nanopipettes clogged within minutes under the same conditions (Figure S6). This difference in biofouling characteristics underscores the significance of aptamers in preventing nonspecific binding to the sensing area.

### Monitoring Endogenous Dopamine Release in Acute Brain Slices

**Figure 5:**
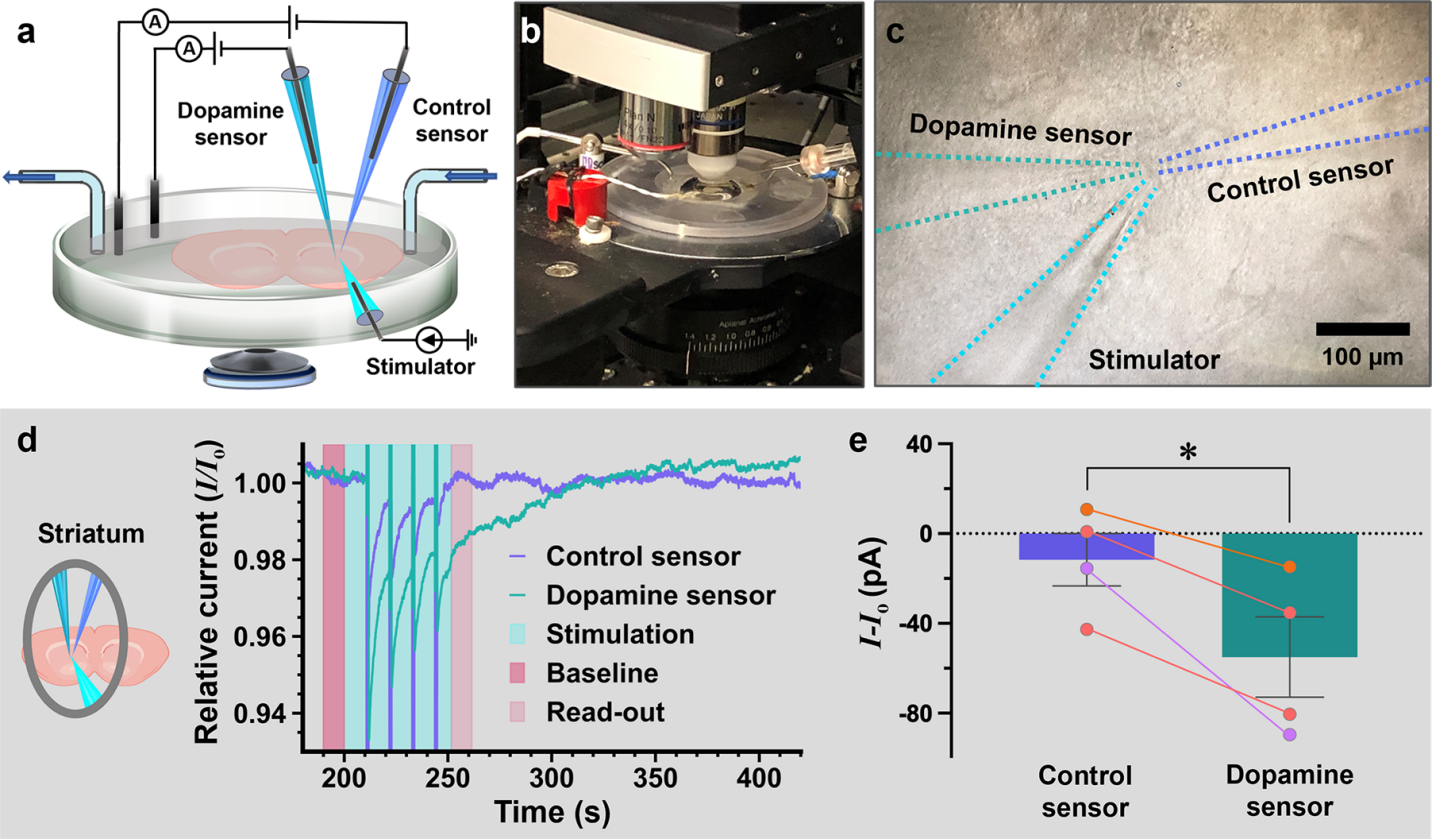
(a) Schematic of the experimental setup for acute brain slice measurements. Dopamine and control sensors are positioned in proximity to a current stimulator. (b) Photograph of the headstage configuration equipped with a microscope. (c) Image of the three capillaries implanted inside the brain slice taken by a CMOS camera. Dotted lines are overlaid to guide the eye to see the approximate locations of the nano- and micropipettes. (d) Simultaneous recordings from the dopamine and control sensors in the dorsolateral striatum relative to the baseline (*I*_*o*_) prior to the 4-train high frequency stimulation (HFS) (in light blue). Regions of 10 s pre and post stimulus (shaded in pink) were used to compare the current reaction. (e) Post stimulus reactions for the control sensor (−11.6 ± 11.7 pA, *N* = 4) and the dopamine sensor (−55.0 ± 17.9 pA, *N* = 4) in the dorsolateral striatum within the 10 s window were averaged [unpaired *t* test: t(6) = 2.027, *P* = 0.0445]. Lines connecting the control and dopamine sensors represent recordings in the same brain slice. Different colors represent measurements in different brains (*n* = 3 animals). All values reported are ± the standard error of the mean.

Aptamer-modified nanopipettes were directly inserted into acute brain slices without the application of positive pressure or solution flow. The nanoscale size of the orifice preoccluded with DNA, likely minimizes the entry of tissue when implanting the sensor. Dopamine aptamer-functionalized nanopipettes and scrambled sequence control sensors were positioned in the brain slice within ∼50–100 μm from a high frequency current stimulator, employed to evoke dopamine release (Figure 5a). The three capillaries were positioned in close proximity and in a similar arrangement for each measurement, so that the specific and control sensors experienced comparable environmental variations and endogenous dopamine release. The positioning of the two nanopipettes within the brain slice was aided by a microcontroller and bright-field microscope headstage (Figure 5b). The stimulator was first inserted, and then, the two sensors were gradually brought into the field of view (Figure 5c).

The measurements were performed in the dorsolateral striatum, which receives dense innervation from the substantia nigra pars compacta of the brainstem. This brain area has been assessed for dopamine release using FSCV.^34,35^ Initially, to test the feasibility of aptamer-modified nanopipettes for dopamine detection in brain slices, a strong high frequency stimulus (HFS) was employed in the dorsolateral striatum to maximize dopamine release (4 trains, 100 Hz, 9 mA stimulus). In repeated measurements, we observed that the 9 mA stimulus inducing brain contractions caused movement and subsequent misalignment of the inserted capillaries. To this point, we decreased the stimulus to 300 μA, a current amplitude used to evoke dopamine release for patch-clamp recordings and FSCV measurements (Figure S7a).^36−39^ Decreasing the HFS from 9 mA to 300 μA resulted in the detection of smaller current changes from the specific dopamine sensor (Figure S7b).

Employing the 300 μA HFS within the striatum, we observed a decrease in dopamine sensor signal during and post stimulus, while the reference sensor remained relatively stable (Figure 5d). The dopamine sensor regains baseline in under a minute, indicating an autonomous reset likely driven by diffusion-limited access of dopamine to the nanoscale sensor tip and the rapid clearance via reuptake by dopamine transporters. The dynamic *ex vivo* system prevents endogenous dopamine from becoming trapped inside the aptamer-modified sensor. Conversely, in static *in vitro* systems where dopamine can accumulate inside the aptamer-occluded nanopore, a reset protocol is imperative for sensor reuse. While prior real-time measurements were conducted at an applied potential of 0.5 V, where maximal response to dopamine was recorded in the *I*–*V* curves, hardware limitations of the patch-clamp setup restricted the applied potential window between ±0.2 V, likely resulting in a smaller signal response. Nevertheless, a statistically larger decrease was observed for the dopamine sensor vs the control sensor over four individual slices originating from three different animals (Figure 5e).

The same process was repeated in the cortex, which receives inputs from dopaminergic cells of the ventral tegmental area.^40^ A comparable sensor and control signal behavior was observed in the cortex as in the striatum: the reference current remains stable, while the first HFS results in a decrease in current response during and after the stimulation. The recordings are shown in Figure S8a were specifically conducted within the same brain slice with the dopamine and control sensors deployed in the striatum (Figure 5d). Multiple usage of the same dopamine and control sensors demonstrate retention of sensor functionality despite implantation in tissue. Not all measurements in the striatum and cortex were conducted in the same brain slice to ensure that the effects observed were not due to the degeneration of the tissue or other effects induced by the HFS. Measurements in the cortex were repeated in two different animals for a total of four dopamine sensor recordings and two control sensor recordings, which resulted in statistical differences (Figure S8b).

Despite statistically relevant differences between specific vs control sensors, precise quantification of the released dopamine remains a challenge. Upon sensor implantation, a minor shift in baseline current occurs due to changes in the environmental resistance. This baseline change complicates the correlation of values from sensor precalibration *in vitro* to recorded values within tissue environments *ex vivo*. A measurement protocol that combines both pre- and postexperimental calibration may offer a viable solution to enable accurate quantification of endogenous small-molecule release inside environments in which calibration cannot be conducted. Additionally, variability between measurements within the same brain region renders biological conclusions regarding dopamine release in different locations challenging. We observed no statistical significance in the HFS-evoked current response measured in the striatum vs cortex (Figure S9). The variability within a test group likely arises from the tissue viability when recordings took longer to obtain stable initial baselines, placement of the dopamine and control sensors, or a combination thereof.

In certain cases, applying a 300 μA HFS did not elicit a measurable change in the current response in both dopamine and control sensors (Figure S10). However, these measurements indicate that the HFS itself does not alter the sensor signal. If the sensors, sensitive to nanoscale volumes, are not positioned in adequate proximity to the stimulator, the encounter of dopamine with the nanopore is diffusion limited. Negligible current changes are also observed when the same brain tissue location is stimulated a second time, aligning with the fact that HFS depletes the local dopamine store (Figure S11). As dopamine can either be reuptaken or washed away by the constant medium flow, sensors placed too far from the stimulator will not experience the local dopamine release. The placement of the nanopipettes is challenging, and the positioning consistency cannot be guaranteed due to microscope limitations.

To address this positioning challenge in the future, we will couple our nanopipette sensors as probes to a scanning ion conductance microscope, which would enable simultaneous topographic and chemical mapping of neurons. This methodology holds the potential to allow precise nanopipette placement with nanoscale resolution and even opens the possibility of approaching synapses. Further, we are currently strategizing the integration of the specific and control sensors into one capillary to overcome this issue. A double barrel configuration that separates two sensing chambers by ∼20 nm^41^ would alleviate positioning challenges while reducing the spatial distance between the two sensors by orders of magnitude, a critical factor for differential measurements in complex milieu. Herein, we demonstrated the possibility to implant selective and sensitive dopamine aptamer-modified nanopipette sensors inside tissue without clogging the nanopore. We envision that innovative nanopipette configurations will enable neurotransmitter recordings with high sensitivity and spatial resolution near neuronal networks *in vitro* or in specific brain areas *ex vivo*.

## Conclusions

Dopamine aptamer-modified nanopipettes were deployed as novel nanotools to enable measurements of endogenous dopamine release from human dopaminergic neurons cultured *in vitro* and from specific brain regions upon electrical stimulation *ex-vivo*. The high selectivity of the dopamine sensors in the presence of catecholamines that are traditionally challenging to differentiate such as norepinephrine and L-DOPA was confirmed in neuronal media prior to dopamine detection from dopaminergic neurons and acute brain slices. Differential measurements using control sensors deployed in parallel to the dopamine sensors ensured that observed changes in the current responses were dopamine-specific rather than variations in pH or ionic content in biological systems. Compared to existing implantable neurotechnologies, advantages of the nanopipettes with ∼10 nm orifices include nanoscale spatial resolution and minimized tissue damage upon penetration. Further, despite the nanoscale sensing area, macroscale capillaries allow ease of handling and direct integration into patch-clamp systems, improving translatability of sensors to diverse research groups.

However, to harness the potential of nanoscale resolution, it is important to visualize where the nanopipette tip is located. We envision coupling aptamer-modified nanopipettes to technologies such as scanning ion conductance microscopy that already uses nanopipettes as probes to map topographical features of live cells including neurons.^42−45^ The ionic current through the nanopore is used as feedback to localize the nanopipette from surfaces at nanoscale distances.^46^ Alternatively, locally staining the tissue fluorescently via the implanted nanopipette may enable tip localization. Fluorescent dyes would be injected into the tissue via nanopipettes, owing to electroosmotic flow generated by applying a static potential, the feasibility of which has been reported with single-cell precision.^47^ Optical feedback has the potential to improve the positioning of multiple sensors in complex environments.

Despite various aspects that require further studies and considerations, aptamer-modified nanopipettes have high potential as novel nanotools for neuroscience due to their nanoscale spatial resolution, high sensitivity and selectivity, and reduced surface biofouling compared to state-of-the-art neurochemical analytical methods. In the future, to improve our understanding of the role of dopamine in modulating transmission, dynamic dopamine flux should be monitored in highly localized regions, requiring sensors with submicrometer dimensions. Electrodes used for FSCV^9,10,48^ as well as implantable neuroprobes^20,49^ with microscale dimensions cannot approach nanoscale-level synaptic sites.

Finally, beyond dopamine sensing, a significant advantage of these nanoscale sensors is their generalizability. Hypothetically, any small molecule for which specific aptamers can be isolated and which undergo a conformational change can be integrated into the established fabrication and chemical modification protocols of aptamer-functionalized nanopipettes. The adaptability of this technology opens diverse possibilities for probing fundamental neurotransmitter dynamics in complex systems. Measuring neurochemicals that serve as key modulators in psychiatric and neurodegenerative diseases may lead to improved strategies to monitor, manage, and potentially treat such brain disorders in the future.

## Materials and Methods

### Materials

Sigma-Aldrich Chemie GmbH (Buchs, Switzerland) was used as the chemical supplier, unless otherwise noted. Phosphate buffer saline at 1× concentration (137 mM NaCl, 2.7 mM KCl, 10 mM Na_2_HPO_4_, 1.8 mM KH_2_PO_4_ and pH 7.4 (ThermoFisher Scientific AG, Reinach, Switzerland) was used as received. Artificial cerebrospinal fluid (aCSF) (147 mM NaCl, 3.5 mM KCl, 1 mM NaH_2_PO_4_, 2.5 mM NaHCO_3_, 1 mM CaCl_2_, 1.2 mM MgCl_2_, and 8 mM KH_2_PO_4_) at a pH of 7.3 was prepared in house. All solutions used were prepared with deionized water with a resistivity of 18.2 MΩcm^–1^ produced by a Milli-Q system (Millipore, Billerica, MA). Neurobasal medium was augmented with 2% B27, 1% GlutaMAX, and 1% penicillin streptomycin (all from ThermoFisher Scientific AG, Reinach, Switzerland). Thiolated single-stranded dopamine aptamer: (5′/Thiol/CGA CGC CAG TTT GAA GGT TCG TTC GCA GGT GTG GAG TGA CGT CG 3′) with molecular weight 13,871.8 g/mol, melting point 73.7 °C, and thiolated scrambled sequence: (5′/Thiol/AGT ACG TCG ATG CTC GAT CAG TGG GCT AGG TGC GTA GCG GTC TG 3′) with molecular weight 13,871.8 g/mol, melting point 71.4 °C, were purchased from Microsynth AG (Balgach, Switzerland). All sequences were received in solution (100 μM) after the post-HPLC purification. Until use, DNA solutions were aliquoted and stored at −20 °C.

### Nanopipette Fabrication and Characterization

A laser puller (P2000, Sutter Instruments) was used to fabricate nanopipettes from quartz capillaries (o.d., 1 mm; i.d., 0.5 mm; World Precision Instruments QF100–50–10). For reproducible pulled nanopipettes, the laser puller was heated for at least 1 h before use, and one bare pull (activating a pull without a capillary fastened inside the puller) was performed prior to nanopipette fabrication. To achieve orifices of ∼10 nm, the following parameters were used: (line 1) Heat 750, Filament 4, Velocity 40, Delay 150, and Pull 80; (line 2) Heat 700, Filament 3, Velocity 60, Delay 135, Pull 180. Pipettes within the range of 3.9–4.8 s pull times were used for subsequent functionalization steps. We note that the influence of the pulling parameters appear to vary between instruments and requires fine-tuning and characterization (e.g., using transmission electron microscopy) to ensure nanopore sizes.

### Aptamer Functionalization

DNA aptamers were functionalized on the inside of the quartz nanopipette using a previously reported protocol.^25^ Briefly, vapor phase deposition at 40 °C for 1 h was conducted under vacuum to assemble monolayers of (3-aminopropyl)trimethoxysilane (APTMS) on the nanopipette surfaces. For this procedure, a dry environment must be maintained (<40% humidity). Then, nanopipettes are filled with 1 mM solutions of 3-maleimidobenzoic acid-*N*-hydroxysuccinimide ester (MBS) dissolved in a 1:9 (v/v) mixture of dimethyl sulfoxide and PBS. Liquid is injected into the nanopipettes using MicroFil syringe tips (World Precision Instruments, Sarasota, FL), which must be flushed with Milli-Q water rigorously for dust removal prior to use with chemicals. The 1 h incubation of MBS with the silanized nanopipettes allows the subsequent cross-linking of the amine-terminated silanes to thiolated DNA aptamers.

Simultaneously, aptamers were prepared for functionalization by reducing the disulfide bond serving as protective caps by incubating a 50-fold excess tris (2-carboxyethyl)phosphine (TCEP) relative to aptamer concentration for 1 h at room temperature. The cleaved aptamer solution was then diluted to 5 μM in 1× PBS and cleaned with Zeba spin desalting columns (7K MWCO, 0.5 mL, ThermoFisher Scientific AG, Reinach, Switzerland) to remove unreacted TCEP. Prior to surface attachment, aptamers were denatured at 95 °C for 5 min to eliminate any hybridization or interactions between sequences prior to covalent immobilization and then renatured by cooling rapidly in a cold water bath. Nanopipettes were cleaned once with 1x PBS prior to incubation with the aptamer solution for a minimum of 2 h. Prior to use, the aptamer-incubated nanopipettes were rinsed three times with PBS. During incubation steps, the nanopipette sensors are stored in a moist environment to ensure minimal evaporation of the liquid especially from the nanoscale tip.

### Sensing Measurements via Aptamer-Modified Nanopipettes

Sensor characterization and measurement of the cell culture media were performed with a custom built high gain amplifier. The current was measured between two Ag/AgCl quasi-reference electrodes, one inside the nanopipette (125 μm) and another in the bulk solution (250 μm). Data recordings were performed using a custom written LabVIEW interface (2017, National Instruments), based on the WEC-SPM package provided by the Warwick Electrochemistry and Interfaces Group. Data were collected using an FPGA card PCIe-7852R (National Instruments). Data concerning acute brain slices on the other hand, were acquired via a MultiClamp 700B amplifier controlled by pClamp software (v10.7, Molecular Devices), further specifications can be found in the section concerning brain slice measurements. The current magnitudes and potentials reported in the paper are denoted with respect to the electrode in the bulk solution. The *I*–*V* curves were acquired by sweeping voltage at 0.2 V s^–1^ voltage sweep rates. To avoid dopamine oxidation, 10 wt % of ascorbic acid was added to all prepared dopamine solutions.^50,51^ Sensing measurements were limited to ±0.5 V as higher voltages resulted in signal instability over time and increased noise.

### Regeneration and Storage of Aptamer-Modified Nanopipettes

A minimum of 20 *I*–*V*s were cycled from −0.3 to +0.7 V in 1× PBS to reset the aptamer-modified nanopipette sensors. Such voltage cycles resulted in the release of bound dopamine inside the nanoscale orifice, enabling reuse of the sensor. In *ex-vivo* measurements, hardware limitations only permitted voltage cycling between −0.2 and +0.2 V. However, the continuous renewal of solution via flow facilitated the reset of the sensors. To increase the longevity of the aptamer-functionalized nanopipettes, the sensors were rinsed, filled, then stored with Milli-Q water to reduce etching of the quartz.^52^ Sensors were stored in high humidity environments to prevent evaporation, which could lead to a buffer crystal formation and breakage of the nanoscale tip. Nanopipettes were stored for reuse in centrifuge tubes filled with deionized water at 4 °C.

### Fabrication of Fluidic Platform

The channel mold was designed on Fusion 360 (Autodesk) and then printed using a Phrozen Sonic Mini 4K Resin 3-D Printer with Phrozen Aqua Gray 4k Resin (Phrozen, Hsinchu, Taiwan). After printing, the mold was washed with isopropanol for 20 min and then dried using nitrogen. Subsequently, the print was UV cured for 15 min and baked overnight at 80 °C to ensure minimal solvent residues. The mold was then coated with fluorosilanes (trichloro(1*H*,1*H*,2*H*,2*H*-perfluoro-octyl)silane) for 30 min under vacuum to create a hydrophobic surface and to facilitate subsequent PDMS removal. The polydimethylsiloxane (PDMS) was prepared by mixing 10:1 SYLGARD 184 Silicone Elastomer Base with the curing agent (DOW Silicones Deutschland GmbH, Weisbaden, Germany). The mold was integrated into a Prusia 3-D printed holder, held in place with screws, to facilitate prototyping iterations and PDMS removal (Figure S12). The PDMS mixture was poured into the aforementioned mold and cured at 80 °C overnight. Once cured, the PDMS channel was removed from the mold and glued on a 75 mm × 50 mm glass slide (Corning Inc., New York). To reduce nonspecific binding of molecules to the channels, 2% BSA was flushed through and subsequently rinsed thoroughly with Milli-Q water.

### COMSOL Flow Simulation

COMSOL Multiphysics (version 6.1) was used to devise and validate the macrofluidic channel design. The analysis was of a fully developed laminar flow from two inlets of a Y channel. The aim was to study the flow profile when introducing a slit, needed for the nanopipette to interact with the flowing liquid. This slit was considered as a secondary outlet in the model. A stationary 3-D fluid flow model was considered with a single phase laminar flow. The channel mold designed on Fusion 360 (Autodesk) was imported into COMSOL. We approximated the density (1000 kg/m^3^) and dynamic viscosity (8.9 ×10^–4^ Pa·s) of the flowing material as water at room temperature. The reference pressure was set to 1 atm. At the inlets we considered a fully developed flow of 1.19 × 10^–8^ m^3^/s, compensation for hydrostatic pressure, suppression of backflow, and normal flow options were applied. Moreover, at the slit, we considered a static pressure of 19.6 Pa due to the hydrostatic pressure of the column of stagnant liquid and compensated for hydrostatic pressure, normal flow, and suppressed backflow options. Finally, physics controlled meshing was used with a fine element size.

### Nanopipette Measurements in Flow

The PDMS channel was mounted on a 3-D printed dish (Original Prusa i3MK3S, Prusa GmbH, Prague, Czech Republic). An Ismatec ISM935 peristaltic pump, with Tygon E-LFL 0.76 mm internal tubing (ISMATEC, Switzerland), was used to supply inlets with PBS and a 100 μM dopamine solution from two independent reservoirs. The continuous flow of liquid filled the channels and then exited the channels passively through the outlet, to be collected in the 3-D printed dish. The custom-built pump removed the liquid from the 3-D printed dish to ensure proper waste removal and to minimize overflowing.

A laminar flow profile was established by using a continuous flow rate of 0.7 mL/min. The sensor was attached to the aforementioned measurement setup, a static bias potential of 0.5 V was applied, and then the nanopipette was positioned and lowered into contact with the PBS via a slit in the PDMS channel. The nanopipette was then lowered a further 2 mm to ensure contact with the flowing liquid, present below the stagnant column of liquid in the measuring conduit. A stable baseline was obtained in PBS for a minimum of 5 min. Subsequently, the nanopipette was moved 3 mm along the slit using a microcontroller, at a speed of 2 mm/s to be positioned in the streamlines of the parallel laminar flowing dopamine solution. A minimum of 5 min was recorded of the nanopipette exposed to the dopamine solution and then transitioned back into the PBS flow to permit reset of the sensor. The reset protocol was required to allow a maximal reset of the sensor.

### Dopaminergic Neuron Cultures

Normal iPSC-derived, human dopaminergic neurons (iXCells Biotechnologies Inc.) were thawed and plated on previously coated poly-l-ornithine-organogel (PLO)/laminin plates at 100k cells per cm^2^ onto a 6-well plate using in the Human Dopaminergic Neuron Maturation Medium (MD-0105-100 ML) for 28 days with 50% medium changes performed every 2–3 days. Media was then collected and sorted at −80 °C. Human motor neurons (iXCells Biotechnologies Inc.) were thawed and plated on previously coated poly-d-lysine (PDL)/matrigel plates at ∼100k cells per cm^2^ onto a 6-well plate using the Motor Neuron Maintenance Medium (MD-0022-100 ML) for 7 days with 50% medium changes performed every 2–3 days. Media was then collected and stored at −80 °C, and the samples were analyzed as quickly as possible post thawing to minimize dopamine degradation.^53^

### Immunostaining

Neuronal cultures were fixed with 4% paraformaldehyde for 15 min at room temperature and then treated with PBS containing 0.1% Triton X-100. After a 15 min PBS wash, cells were blocked with 5% bovine serum albumin in PBS for 1 h and then incubated with the primary antibody in PBS at 40 °C overnight. After a few PBS washes, the cells were stained with secondary antibodies for 1 h at ambient temperature. This process was followed by a 10 min incubation with DAPI and a final round of PBS washes. Primary antibodies used were rabbit anti-Tuj1, (1:500, Covance), rabbit anti-TH (1:500, Pel-Freez), and goat anti-FOXA2 (1:500, R*&*D sytems). Corresponding Alexa Fluor secondary antibodies were then used (1:1000). Stained neurons were imaged with a Cytation 5 Cell Imaging Multimode Reader with a 20× objective.

### Flow Cytometry

Briefly, the cells were dissociated and fixed in 4% paraformaldehyde for 15 min at room temperature. Then, cells were washed twice with ice-cold PBS containing 1% bovine serum albumin, and subsequently 10^6^ cells were incubated with rabbit anti-TH (1:100, Pel-Freez) antibody for 60 min at room temperature. After washing, the cells were incubated with corresponding Alexa Fluor secondary antibodies (1:400) for a further 30 min. After the final washing, the expression of markers was analyzed on a BD FACSLyric flow cytometer. A negative control (isotype, no primary antibody added) was used to gate samples.

### Brain Slice Measurements

Data were acquired using a MultiClamp 700B amplifier controlled by pClamp software (v10.7, Molecular Devices), filtered at 5 kHz and sampled at 10 kHz (Digidata 1550A, Molecular Device). Animal experiments were approved by the Zurich Cantonal Veterinary Office Zurich. P24–P46, male and female mice were anesthetized by isofluorane and decapitated, and their brains were rapidly transferred to ice-cold dissecting solution containing 110 mM choline chloride, 7 mM MgCl_2_, 25 mM d-glucose, 25 mM NaHCO_3_, 2.5 mM KCl, 1.25 mM NaH_2_PO_4_, 0.5 mM CaCl_2_, saturated with 95% O_2_ and 5% CO_2_. Coronal slices (300 μm thick) were made using a Vibrotome VT 1200S, Leica slicer, then transferred to normal aCSF containing 115 mM NaCl, 3.5 mM KCl, 25 mM NaHCO_3_, 25 mM *D*-glucose, 1.2 mM NaH_2_PO_4_, 2 mM CaCl_2_, 1.3 mM MgCl_2_ and aerated with 95% O_2_ and 5% CO_2_. Slices were kept at room temperature and recovered in aCSF for at least 30 min before recording.

During the recordings, the slices were placed in the recording chamber of an upright microscope and superfused with aCSF kept at 28 °C at a rate of 2 mL/min for continuous oxygenation (95% O_2_ and 5% CO_2_). A CMOS camera (optiMOS, QImaging) was attached to the microscope to visualize the slice and cortical pyramidal neurons or striatal neurons through a computer screen. Aptamer-modified nanopipette sensors and control sensors modified with scrambled sequences were simultaneously used for the voltage-clamp measurements in gap-free mode, and a constant voltage bias of 200 mV was applied throughout the entirety of the recordings.

After the baseline was recorded for 10 min, a high-frequency stimulation (HFS) of 300 μA was applied. To induce HFS, four 100 Hz trains were repeated for a 1 s duration every 10 s while maintaining the voltage constant at 200 mV during the entirety of the stimulation protocol. The current was recorded for a minimum of 10 min following HFS stimulation. The stimulation (300 μA, 0.1 ms) was performed through a borosilicate glass pipet (1.5 OD × 0.86 ID × 75 L mm, Harvard Apparatus) filled with aCSF, and connected to a constant current stimulator (Stimulus Isolator Model IS4 Primelec). The two sensors and the stimulator were placed within 50–100 μm of each other. All sensors were reset before and after the measurements by applying voltage sweeps between −200 to +200 mV for a minimum of 20 cycles until reaching a stable current. Although the sensors typically reset during the real-time recording conducted in flow, the reset protocol ensures that any remaining dopamine confined in the nanopore is ejected prior to reuse. During the experimental time frame of a few hours, evaporation from the top of the nanopipette is negligible (Figure S13).

The baselines of both dopamine and reference sensors were normalized with respect to their baseline currents for comparative representation. The average of the current prior and post stimulation was calculated for both sensors within a 10 s window.

### Statistics

All statistics were carried out using GraphPad Prism Version 9 (GraphPad Software Inc., San Diego). Data are reported as means ± standard errors of the means with probabilities *P* < 0.05 considered statistically significant. Comparative data were evaluated by either one-way analysis of variance followed by Tukey’s multiple group comparisons or Student *t* tests.

## Supporting Information Available

Dopamine concentration-dependent measurements and control measurement in ascorbic acid (*I*–*V* curves) (Figure S1); Concentration-dependent real-time response (Figure S2); resetability of dopamine sensors (Figure S3); flow cytometry measurements of human iPSC-derived dopaminergic neurons (Figure S4); pH values of media received from iXCell Biotechnologies, Inc. (Figure S5); clogging of bare nanopipettes in cell media (Figure S6); additional data of sensors interfaced with brain tissue (Figure S7−S11); macrofluidic mold fabrication (Figure S12); evaporation of liquid from nanopipettes (Figure S13). (PDF)
