# Supplementary material for: Interfacing Aptamer-Modified Nanopipettes with Neuronal Media and Ex Vivo Brain Tissue
Source: ACS Meas Sci Au. 2023 Nov 22;4(1):92–103. doi: 10.1021/acsmeasuresciau.3c00047 (PMC10885324; doi:10.1021/acsmeasuresciau.3c00047)
Supplement: Supplementary file 1 — tg3c00047_si_001.pdf [file tg3c00047_si_001.pdf]

Supporting Information

Interfacing Aptamer-Modified Nanopipettes with Neuronal Media and *Ex Vivo* Brain Tissue

Annina Stuber,[1] Anna Cavaccini,[2,3] Andreea Manole,[4] Anna Burdina,[1]
Yassine Massoud, [1] Tommaso Patriarchi,[3,5] Theofanis Karayannis,[2,3] and Nako Nakatsuka[1*]

[1]*Laboratory of Biosensors and Bioelectronics, Institute for Biomedical Engineering, ETH Zürich, CH-8092, Switzerland*

[2]*Laboratory of Neural Circuit Assembly, Brain Research Institute, University of Zurich, CH-8057, Switzerland*

[3]*Neuroscience Center Zurich, University and ETH Zurich, CH-8057, Switzerland*

[4]*iXCells Biotechnologies, Inc., San Diego, CA, 92131, U.S.A.*

[5]*Institute of Pharmacology and Toxicology, University of Zurich, CH-8057, Switzerland*

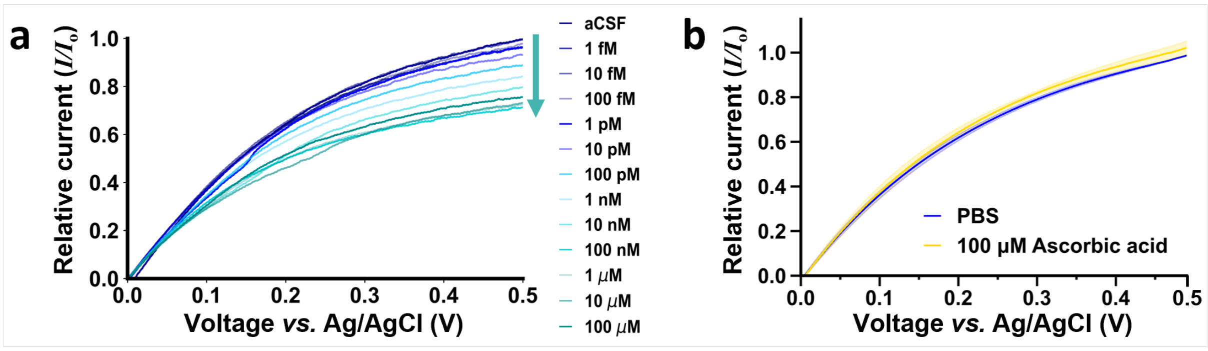

Figure S1. Dopamine aptamer-modified nanopipettes sense dopamine specifically in artificial cerebrospinal fluid (aCSF). (a) Cyclic voltammograms show the concentration-specific decrease in current response from the aCSF baseline with increasing amounts of dopamine. The curves are represented relative to the aCSF baseline measurement at 0.5 V. (b) Cyclic voltammogram measurements of phosphate buffered saline (PBS) baseline and upon addition of 100 µM ascorbic acid. A total of *N*=3 nanopipettes were averaged, indicated by the solid line, and reactions are represented relative to the current measured at 0.5 V in PBS. The standard error of the mean is represented by the shaded area around the solid line. The 100 µM ascorbic acid concentration used herein, is 10-fold higher than the 10 µM ascorbic acid added when measuring 100 µM dopamine (10 % wt).

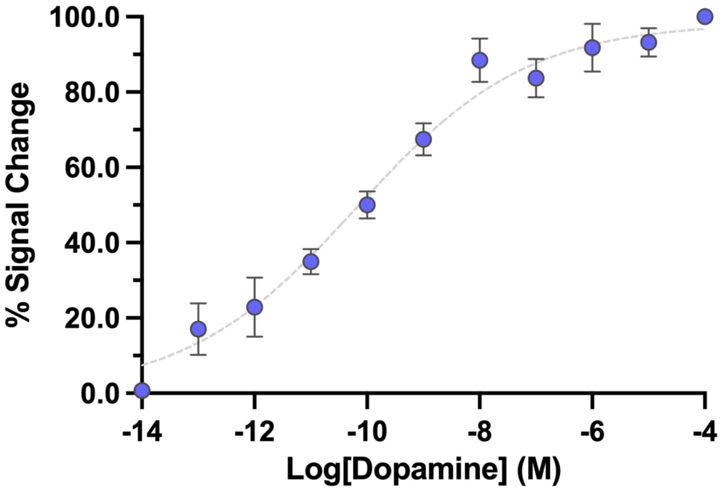

Figure S2. Concentration-dependent real-time response in artificial cerebrospinal fluid (aCSF). The calibrated response of *N*=3 dopamine sensors normalized to the maximal saturated current response at a concentration of 100 μM. The sensors were exposed to range of dopamine concentrations in aCSF measured under an applied static bias of 0.5 V.

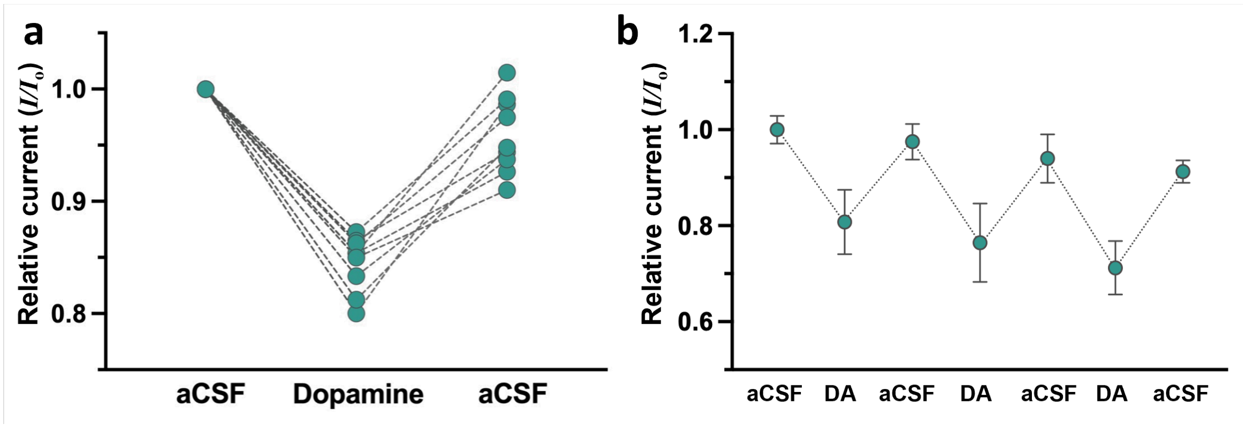

Figure S3. Resettability of dopamine sensors in artificial cerebrospinal fluid (aCSF). (a) After exposure of dopamine aptamer-functionalized nanopipette sensors to high concentrations of dopamine to saturate the sensor (100 μM), the sensors were reset in phosphate buffered saline (PBS), and then retested in aCSF for N=8 sensors. Through allosteric effects with divalent cations, which are present in aCSF but not in PBS, dopamine binding is stronger in aCSF.[1] Thus, conducting rinsing cycles in PBS appeared to improve the resettability of the sensors. (b) A single dopamine sensor can be deployed multiple times in aCSF upon exposure to dopamine and then reset by performing voltage sweep protocols (N=3). All values are represented relative to the baseline.

Reference:

1. Nakatsuka, N.; Abendroth, J. M.; Yang, K.-A.; Andrews, A. M. Divalent Cation Dependence Enhances Dopamine Aptamer Biosensing, *ACS Appl. Mater. Interfaces*, 13, 9425–9435, 2021.

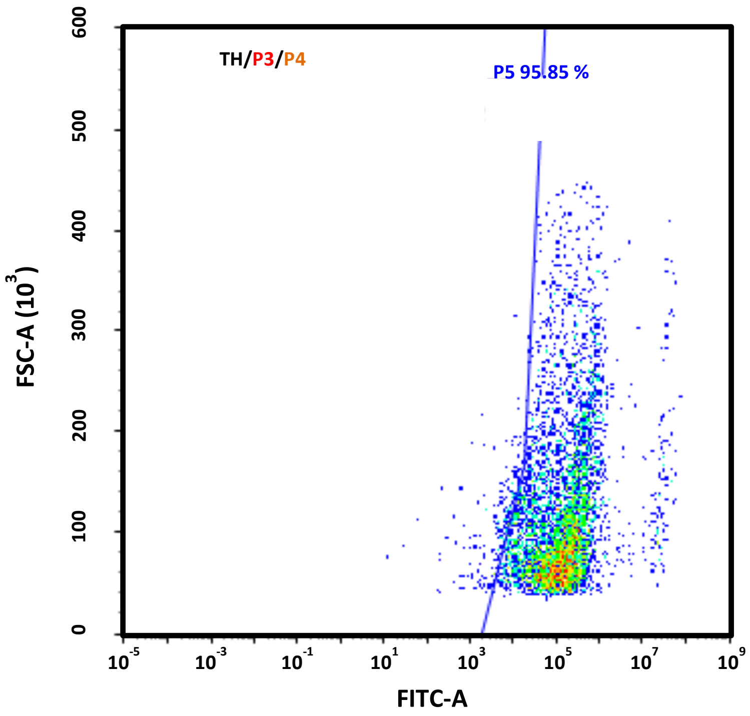

Figure S4. Flow cytometry measurements of Human iPSC-derived dopaminergic neurons.

Flow cytometry measurements demonstrated a highly specific population of fully differentiated, TH-positive midbrain dopaminergic neurons.

Photo	Sample	pH
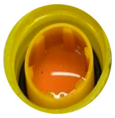	Human dopaminergic neuron maturation medium	7.75
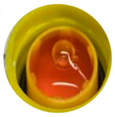	Negative control medium<br>7 day incubation of motor neurons	7.84
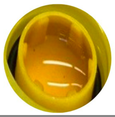	Human dopaminergic neuron medium<br>30 day incubation of dopaminergic neurons	7.42

Figure S5. Variation in pH between different media received from iXCell Biotechnologies, Inc. Table of various media tested: maturation media with no prior cell contact, media collected from negative control motor neuron cultures, and media collected from dopaminergic neurons. Different colors suggested a varied pH value, which was subsequently measured with a pH meter.

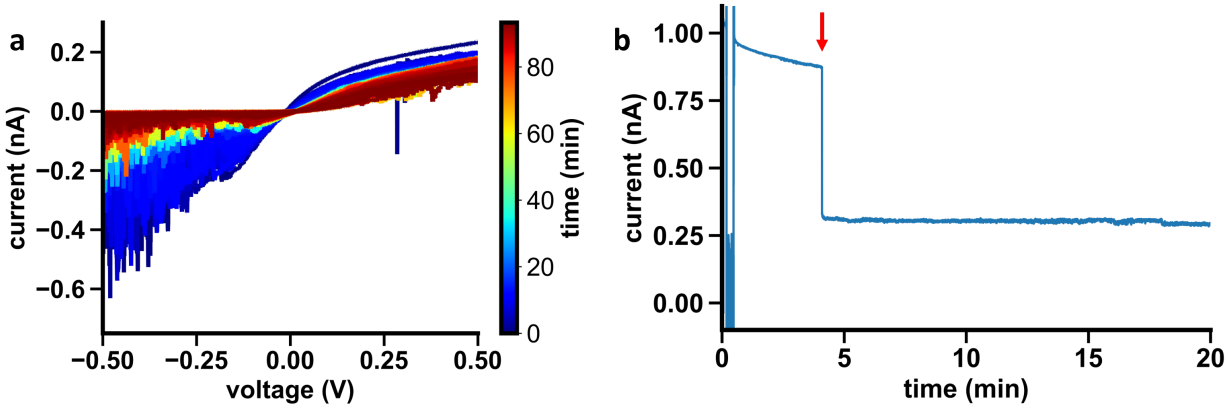

Figure S6. Bare nanopipettes clog in neurobasal medium. (a) The potential was swept between -0.5 to +0.5 V for over 90 minutes, while the ionic current was monitored. The current-voltage traces are overlayed and the progression over time is represented by the change in color. Within minutes, the nanopores begin to be clogged, observed by a decrease in the measured current. (b) A static bias potential of 0.5 V was applied similarly to real-time measurements, and the current dropped from a starting current of ~1 nA to ~0.3 nA after less than 5 minutes of exposure to neurobasal medium, indicated by the red arrow.

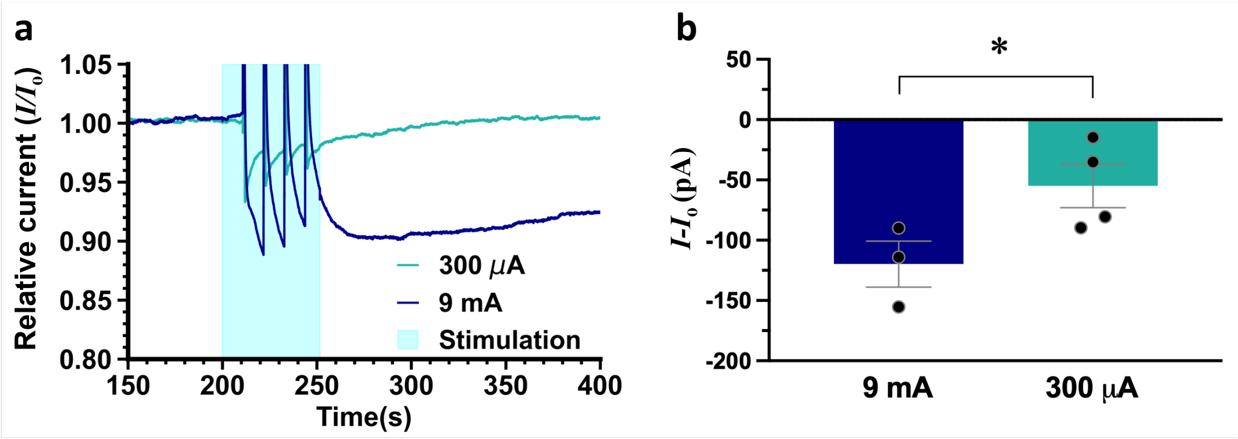

Figure S7. Variations in dopamine release in murine dorsolateral striatum based on stimulation amplitude. (a) Overlay of dopamine sensors reacting to 9 mA *vs.* 300 µA 4-train high frequency stimulations (HFS) in the dorsolateral striatum. Both signals are represented relative to baseline prior to the HFS. (b) A statistically significant difference in sensor signal was observed, where 9 mA HFS (-119.8 ± 19.1 pA) showed larger changes in the current response *vs.* 300 µA (-55.0 ± 17.9 pA) [unpaired t-test: t(5)=2.440, *P*=0.0293].

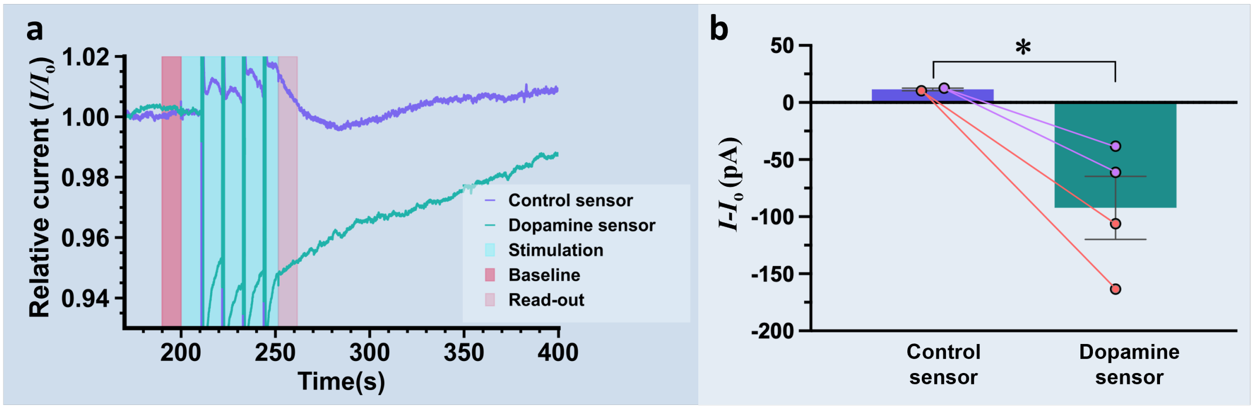

Figure S8. Ionic current measured in real time in murine cortex of both dopamine-aptamer functionalized nanopipettes and control sensors. (a) Real-time recordings of the dopamine and control sensors in the cortex relative to the baseline prior to the HFS (section highlighted in dark pink). (b) Control (11.5± 1.2 pA, N=2) vs. dopamine (-92.2 ± 27.6 pA, N=4) sensor measurements were compared in cortical slices from n=2 animals [unpaired t-test: t(4)=2.504, P=0.0332] averaged withina 10 s window post stimulus (region represented in light pink**).**

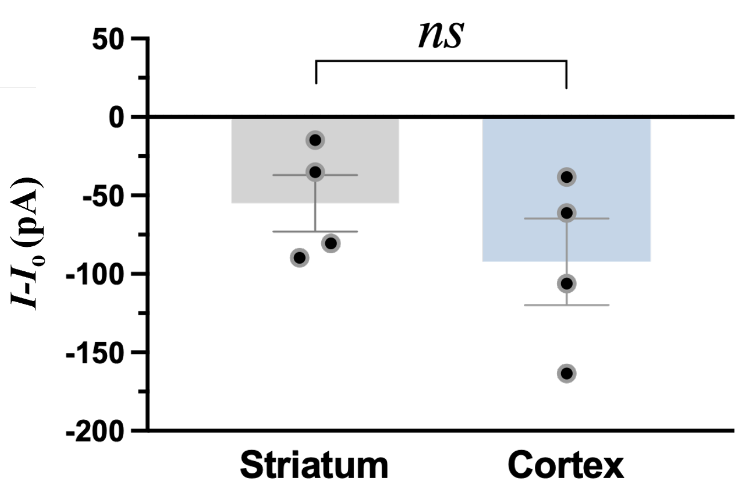

Figure S9. Comparison of the dopamine sensor response upon high frequency stimulation (HFS) in the dorsolateral striatum *vs.* cortex. Values extracted for the change in the current response, taken as the average of the 10 s window post HFS, was not statistically significant (*ns*) between the striatum (-55.0 ± 17.9 pA) *vs.* the cortex (-92.2 ± 27.6 pA) for N=4 measurements in each location [unpaired t-test: t(6)=1.132, P=0.1505].

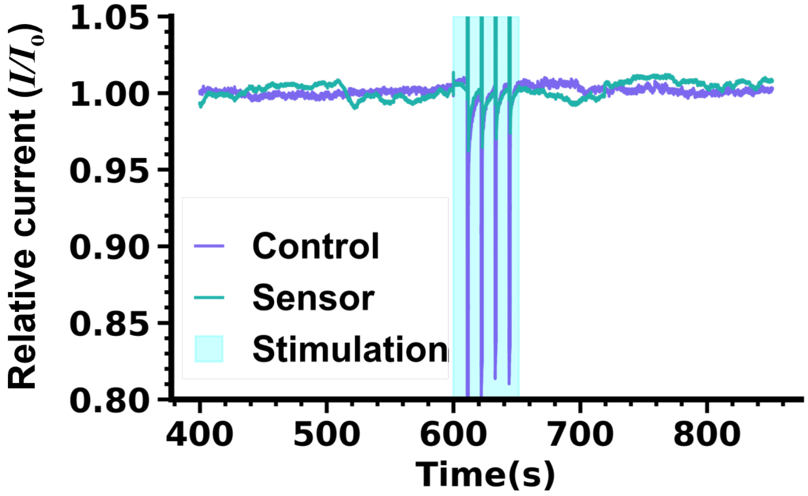

Figure S10. Challenge of locating sensors with adequate spatial proximity to the stimulator for dopamine detection. In some measurements, post-300 µA high frequency stimulation (HFS) in the dorsolateral striatum, negligible signal reaction was detected by both the dopamine and control sensors. Such results indicate the importance of localizing the specific and control sensors to the point of stimulation. Signals are represented relative to the baseline recording.

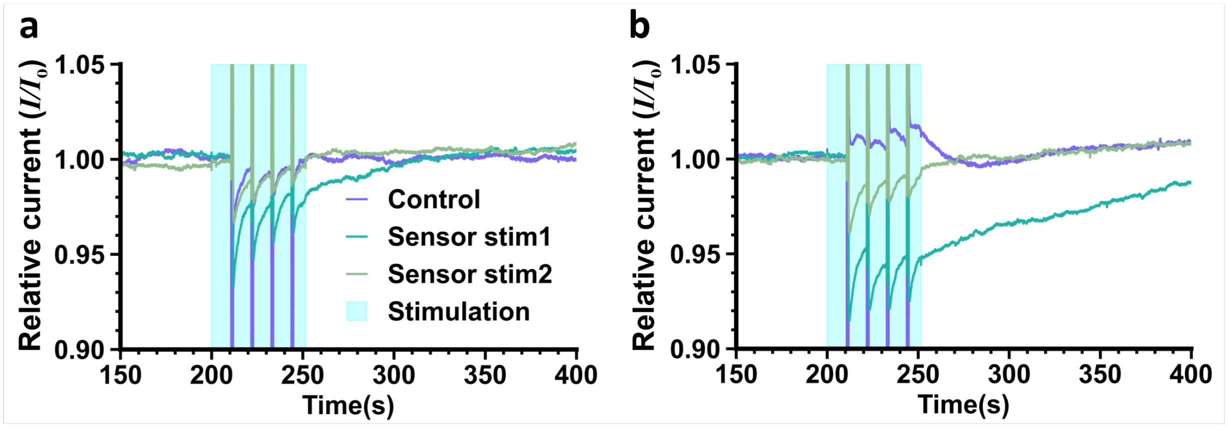

Figure S11. First *vs.* second high frequency stimulation (HFS) comparisons. Traces of ionic currents detected by the sensor in response to both first *vs.* second stimulations are overlaid. The nanopipettes are reset between the two stimulations *in situ*. The nanopipettes and stimulator are not moved between the first and second stimulation (a) in the striatum and (b) in the cortex. All traces are represented relative to their respective baseline measurements.

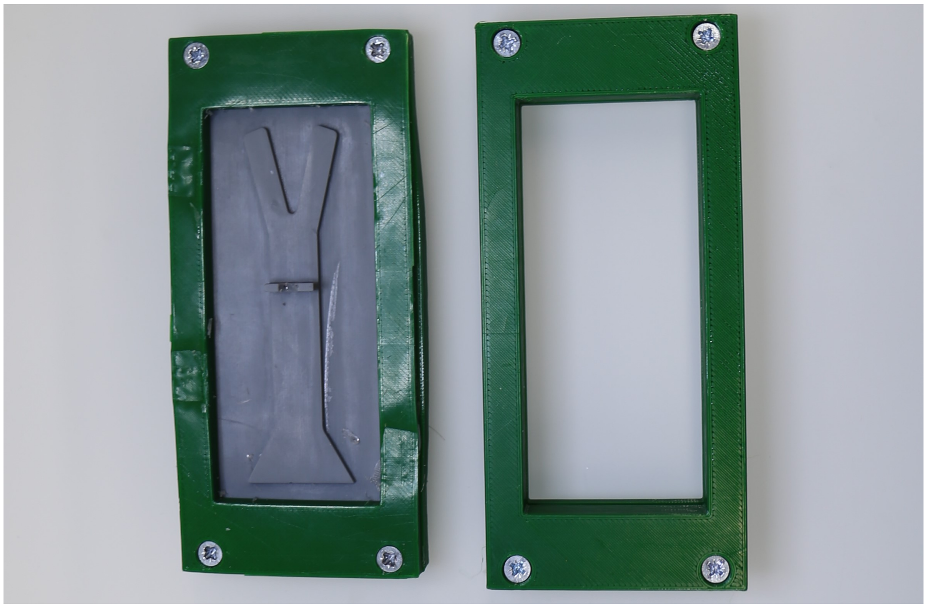

Figure S12. 3-D printed macro-fluidic channel mold. Photograph of the 3-D resin printed macro-fluidic channel mold (grey) mounted in a PLA 3-D printed holder (green). The holder is then screwed together and PDMS is poured on top of the grey mold and set to cure overnight at 80 °C. The 3-D printed holder is used to allow both facilitated PDMS removal, as well as faster prototyping iterations.

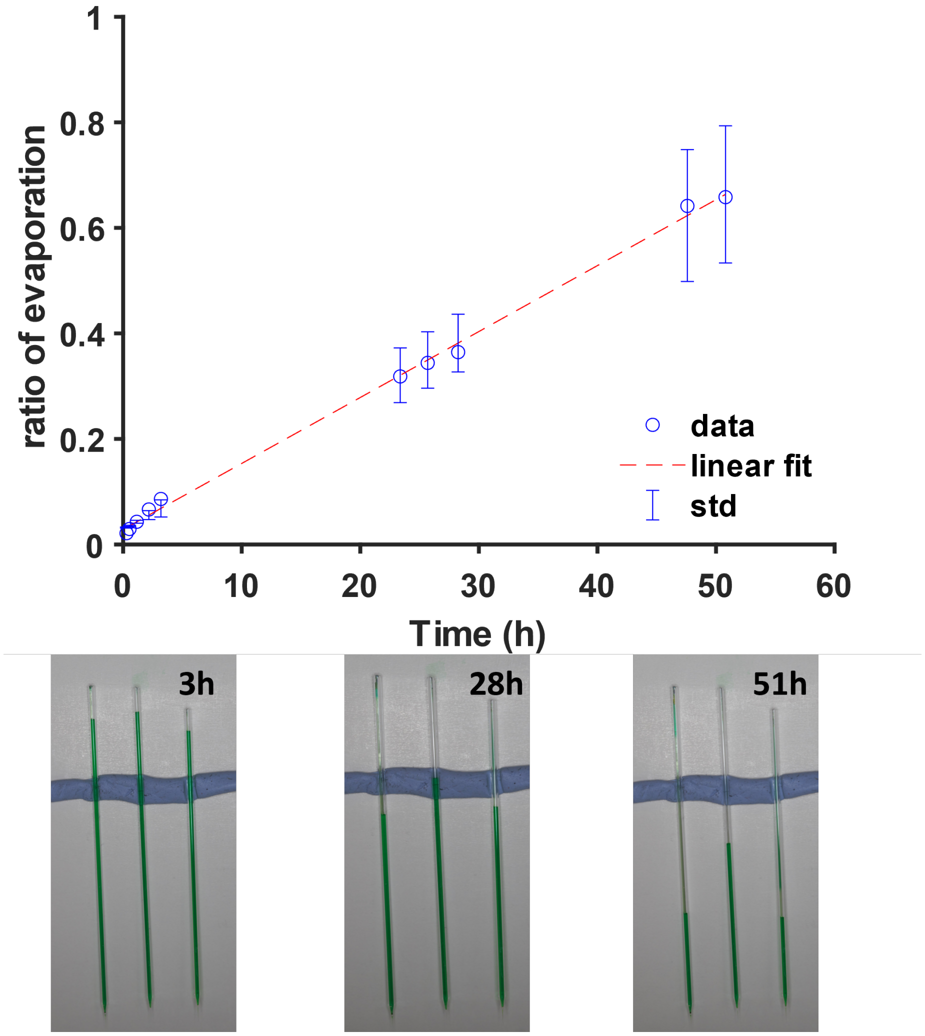

Figure S13. Evaporation of liquid inside the nanopipette over time. Food coloring was added to the liquid injected inside the pipettes and allowed to evaporate at room temperature for over 2 days. The ratio of evaporation was identified by measuring the change of visible liquid height with respect to the entire pipette length, using ImageJ. Photos were taken every few hours, and values represented on the graph were averaged over 3 pipettes subject to the same conditions.
